# Birthing parent perspectives on measuring the quality of perinatal care: metrics, timing, and process

**DOI:** 10.3389/frhs.2024.1473848

**Published:** 2024-12-10

**Authors:** Kristin P. Tully

**Affiliations:** Department of Obstetrics and Gynecology, School of Medicine, University of North Carolina at Chapel Hill, Chapel Hill, NC, United States

**Keywords:** postpartum, measure, quality, patient-centeredness, respectful care in childbirth, perinatal, obstetric, patient safety standards

## Abstract

**Objective:**

Centering birthing parents is critical for improving reproductive health policies and practices. This study investigates patient perspectives on measuring the quality of perinatal care.

**Methods:**

A cross-sectional qualitative research study was conducted at an academic medical center in the Southeastern United States. Individuals who had recently given birth participated in audio-recorded interviews between May 2020 and September 2020. This analysis addresses the research question, “If we were providing quality healthcare for families, how would we know?” Transcribed and translated responses were inductively coded to develop categories and identify themes.

**Results:**

Forty birthing parents participated in the study. Metrics, timing, and process were identified as important components of meaningfully measuring the quality of perinatal care. Recommended metrics included asking patients whether their health priorities were addressed. Additional metrics of importance were whether coping strategies were provided, the clarity of information provided, patient comprehension of health information, the extent to which care planning was collaborative among patients and their healthcare team members, whether clinicians alleviated patient doubts, patient feelings of being taken care of, healthcare team mannerisms, clinician demonstrations of respect for patient autonomy, and postpartum visit attendance. With regard to timing, patients desired that their healthcare team members “check-in” with them as part of an ongoing, direct dialog. Birthing parents also wanted opportunities to provide feedback soon after encounters. As part of a robust measurement process, they wanted to share their insights with someone who was not a part of their healthcare team, for maintaining confidentiality. The patients desired a “serious platform” with accessible methods for all birthing parents to be able to convey nuanced accounts of their care. They also wanted to hear from the healthcare institutions about their feedback. Birthing parents sought assurances for their perinatal care feedback to be de-identified to protect them from potential retaliation. The participants recognized that they might need to utilize healthcare services from the same institution and individuals in the future.

**Conclusion:**

Birthing parents expressed desire for their perinatal healthcare experiences to be understood. Meaningful quality measurement may be promoted through transparent and multimethod opportunities for patients to securely share insights. In addition to healthcare systems communicating assurances of patient confidentiality, institutional feedback in response to patient-reported experiences is recommended.

## Introduction

Advancing safe, respectful maternity care and positive postpartum experiences is essential (1). The need to cultivate the vital conditions necessary to reduce perinatal mortality and morbidity and to support the thriving of new families is urgent (2). In the United States, the rate of maternal mortality is high, increasing, and disparate (3, 4). Furthermore, many pregnancy-related health complications are preventable (5) and health outcomes vary within and across birthing facilities (6). These childbirth inequities and the neglect of birthing parents through the postpartum period (6) are a call for strengthening healthcare systems. Centering birthing parents is critical for improving reproductive health policies and practices. Yet, existing national-level patient-reported perinatal quality metrics in the United States are not specific to labor, childbirth, or inpatient postpartum care.

The current standard of patient-reported measurement of the quality of perinatal care in the United States is the Hospital Consumer Assessment of Healthcare Providers and Systems (HCAHPS) survey. The 29-item HCAHPS survey is administered to a random sample of patients following their healthcare encounters (8). Survey enrollment and data collection are carried out through third-party vendors, typically by mail. The Centers for Medicare and Medicaid Services requires 100 of the HCAHPS surveys to be completed over four quarters for each hospital to receive a star rating (9). There is no HCAHPS survey or other national-level patient-reported quality care measure specific to perinatal care services. Furthermore, there are no requirements for calculating the percentage of perinatal patients who complete HCAHPS surveys or for carrying out survey completion among patients with limited proficiency in the English language.

Listening to diverse patients and following through on their priorities is key to providing respectful, equitable, and supportive healthcare services. The 2022 release of the White House Blueprint to Address the Maternal Health Crisis included the proposed establishment of a Birthing Friendly Hospital designation. This initiative was framed as part of “ensuring those giving birth are heard and decision makers” in quality obstetric care. The designation is intended to increase patient–families awareness of “which hospitals are taking steps to provide high-quality care” and for hospitals to be “more accountable for the quality of care they provide.” However, the designation metrics currently address only two items: first, whether a hospital participates in a statewide or national perinatal quality improvement collaborative program and second, whether the hospital implements evidence-based quality interventions to improve maternal health (10). Despite the importance of structuring healthcare services to address what birthing parents need to know, feel, and have happen to be safe and well (11), little is known about their perspectives on how the quality of labor, childbirth, and inpatient postpartum care might be measured. To inform initiatives such as the Birthing Friendly Hospital designation and HCAHPS, this study investigated patient perspectives on measuring the quality of perinatal care.

## Methods

The research team conducted a cross-sectional qualitative study with birthing parents as part of a large mixed methods research project, the Postnatal Patient Safety Learning Lab. The study setting was an academic medical center in the Southeastern United States. Approximately 4,000 births per year are reported at the hospital. Patients who birth at the hospital are racially and socioeconomically diverse. They also have a wide range of medical acuity levels. The patient population ranges from low-risk birthing families cared for by certified nurse midwives to complex referral cases with multiple maternal and fetal comorbidities and supported by maternal fetal medicine physicians. In the postnatal unit, nurses admit maternal and infant patients and the healthcare team is then responsible for monitoring, treating, and providing information to postpartum patients. The postnatal unit stay includes daily rounds and nursing assessments. Interpretation services on labor delivery and the postnatal unit include in-person Spanish interpreters, as well as video and telephonic language lines. Prior to postpartum hospital discharge, clinicians provide patients with verbal and written health and safety information. This content includes health warning signs and information on clinical appointments.

**Figure 1:**
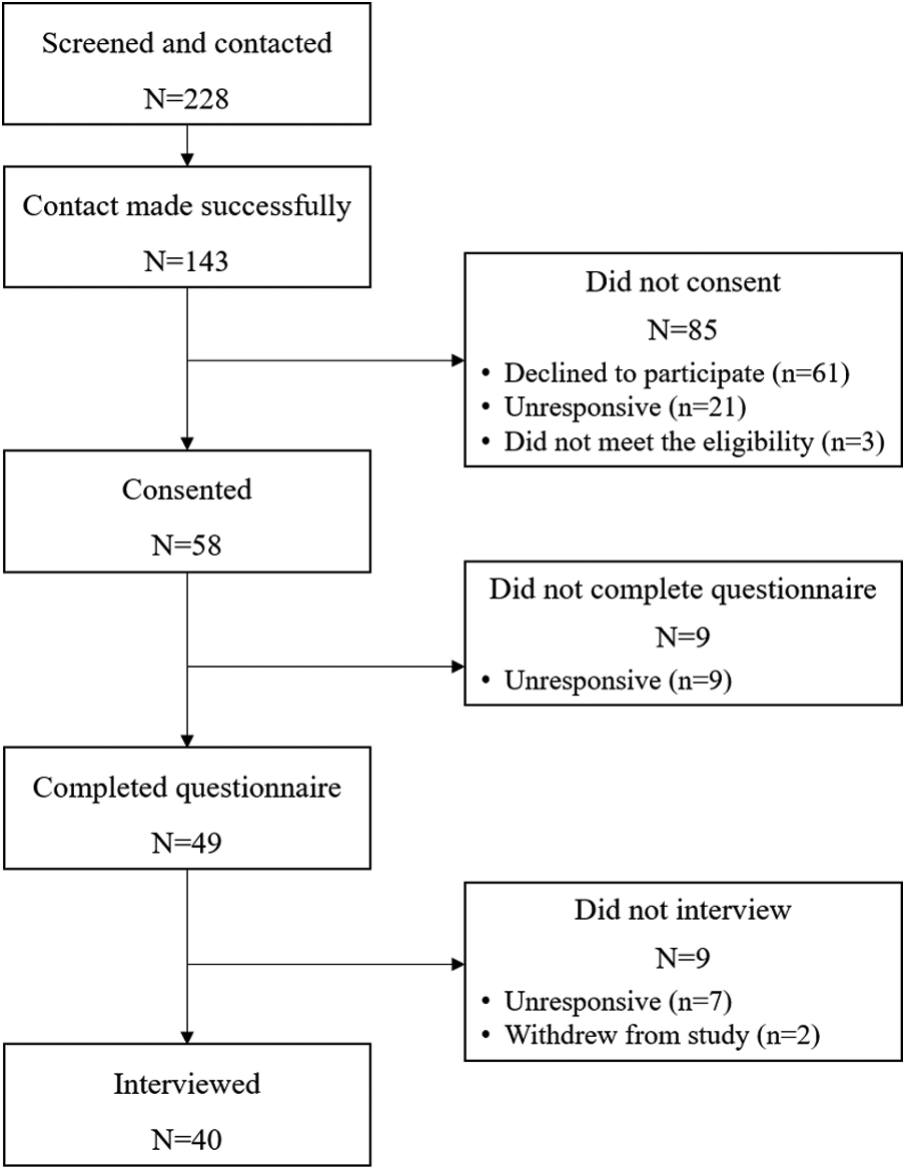
Participant recruitment, enrollment, and study completion.

Participant recruitment is illustrated in Figure 1. Following University of North Carolina at Chapel Hill Biomedical Institutional Review board approval #19-1900, the study team members identified potential postpartum participants at a hospital in the Southeastern United States between May 2020 and September 2020 using electronic medical records and clinician referrals (clinicians provided study enrollment information to potential participants). Individuals were eligible if they were at least 18 years old, the birthing parent of a live-born singleton or twins, less than 2 weeks postpartum at recruitment, spoke English or Spanish, had access to a phone or computer, and were discharged to a residence. The exclusion criteria included individuals with a preferred language other than English or Spanish, who were currently incarcerated, or whose infant(s) had planned placement for adoption.

A bilingual native-speaking (Spanish/English) research assistant contacted 143 individuals through convenience sampling. A total of 58 individuals consented to participate (*n* = 61 declined, *n* = 21 were unresponsive to follow-up, and *n* = 3 did not meet the full study inclusion criteria). Of these, 49 individuals completed a study questionnaire in the days prior to their telephone interview. Seven participants completed the questionnaire but could not be reached for an interview after two contact attempts. Two participants withdrew from the study (one was voluntary withdrawal and the other was ineligible). The participants were sent a $20 gift card electronically or by mail following the completion of the questionnaire. An additional $20 gift card was sent to those who completed the interviews, for a total of up to $40 each.

The semistructured interviews were conducted over telephone by team members who were not involved in patient care. The researchers were trained in qualitative methods and were either native English or Spanish speakers. Interview questions explored birthing parents’ perspectives on their experiences with perinatal healthcare. The interviews were audio-recorded, with professional human translation and transcription. The study team members compared audio files with the transcriptions to check for accuracy. Transcriptions were entered into a spreadsheet verbatim, organized by interview question and participant study identification number. To get familiarized with the data, the author read all responses to the interview question of interest in this analysis: “If we were providing quality healthcare for families, how would we know?” Then, the author conducted thematic content coding (12). Similar keywords and phrases in the transcriptions were inductively coded. *In vivo* coding used words from the data as codes, such as “how are you coping?” The codes also described the attributes of the data, such as the descriptors “healthcare team practices” and “patient experiences.” The codes were grouped into categories and then the author developed themes relevant to guiding future action. This analytic process entailed repeated review of quotes, codes, and categories with memos and comparisons throughout the process for refinement.

## Results

Forty birthing parents completed an interview. Most of the participants gave birth ≥37 + 0 gestational weeks (95.0%) and had a vaginal delivery (60.0%). They primarily identified themselves as Hispanic (43.6%) or non-Hispanic Black (30.7%). The interview was conducted in Spanish with 40.0% of the participants. Participant characteristics are listed in Table 1.

**Table 1:** Characteristics of the 40 birthing parent–infant participants.

	*n* (%)
Birthing parent ethnicity and race	
Non-Hispanic Black	12 (31)
Non-Hispanic white	4 (10)
Hispanic	17 (44)
Asian	3 (8)
Native American	2 (5)
Multiple ethnicity and race	1 (3)
Birthing parent age	
18–24 years old	8 (21)
25–34 years old	22 (56)
35 years or older	9 (23)
Language(s) spoken at home	
English	29 (73)
Spanish	11 (33)
Other	2 (1)
Baby's gestational age at birth	
<34 weeks	1 (3)
34 + 0 to 36 + 6 weeks	1 (3)
37 + 0 to 39 + 6 weeks	29 (73)
40 weeks or more	9 (23)
Type of birth	
Vaginal	24 (60)
Cesarean section	16 (40)
Baby received care in the neonatal intensive care unit (NICU)	
No	31 (84)
Yes	6 (16)

The one participant who was categorized as multiple ethnicity and race selected non-Hispanic, Black, white, and Native American.

**Figure 2:**
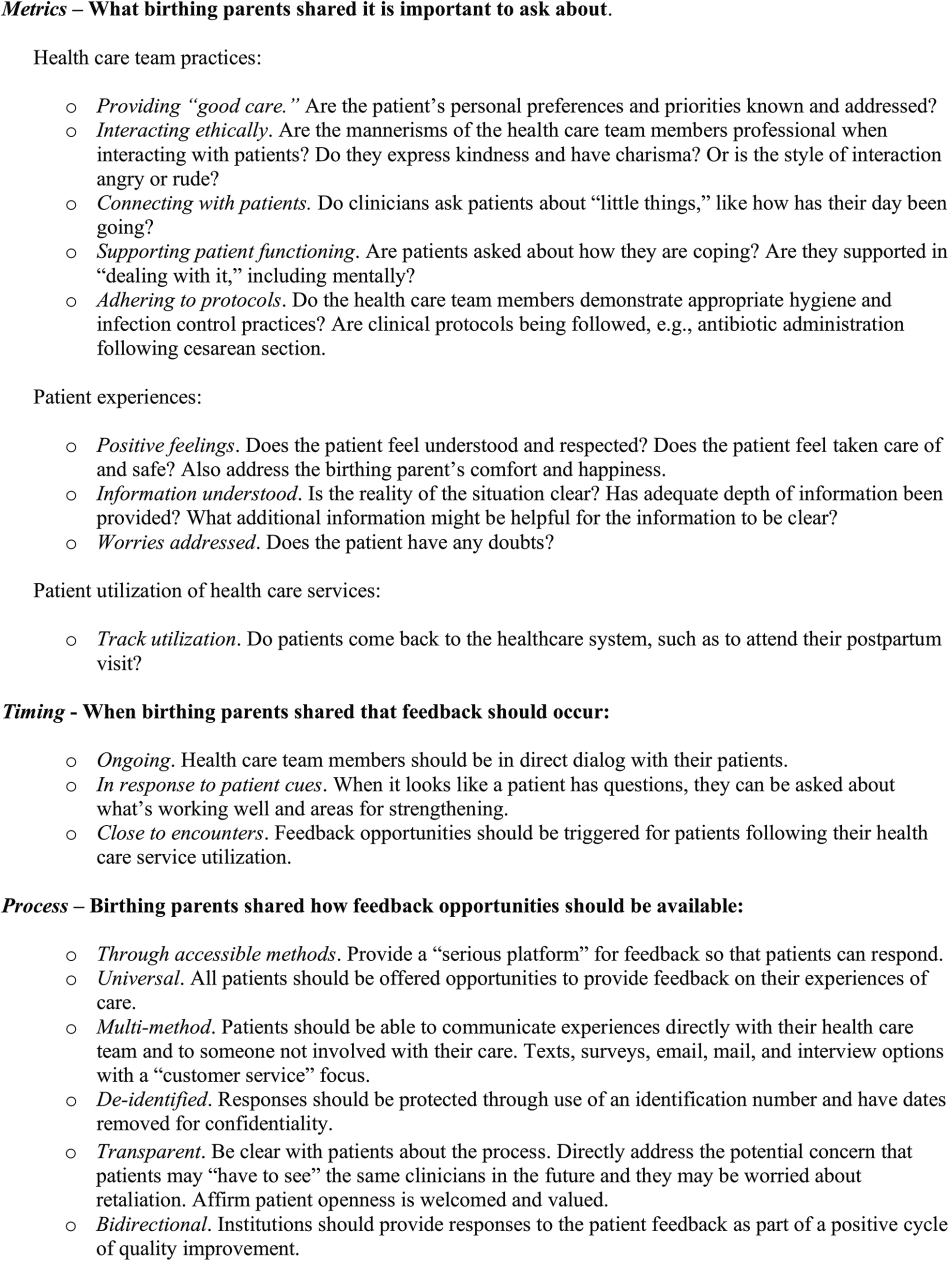
Birthing parent perspectives on measuring the quality of perinatal care. Recommendations for metrics, timing, and process.

In response to the interview question, “If we were providing quality healthcare for families, how would we know?”, metrics, timing, and process were identified as themes. Although a few participants said that they did not know about the quality of healthcare provided or that the healthcare system was good as it is, most of the birthing parents gave their perspectives about measuring the quality of perinatal care in relation to healthcare practices, patient experiences, and service utilization (*Metrics*), patients having ongoing opportunities to offer feedback (*Timing*), and having a robust, universally accessible, and confidential feedback system (*Process*). A patient summarized these interrelated components of measuring quality care thus: “I think always asking how we're doing, how we're feeling, what we need” (M37, Hispanic, Spanish-speaking birthing parent). The findings are described by theme below with illustrative quotes. Figure 2 presents a list of the results.

### Metrics—healthcare team practices, patient experiences, and patient utilization of subsequent healthcare services as indicators of quality care

Recommended performance metrics of healthcare team practices addressed patient priorities. The comprehensiveness and timeliness of care was important: “Did you get everything you need during the time that they're there?” (M22, non-Hispanic Native American birthing parent). The participants also highlighted the importance of healthcare team members having positive mannerisms when interacting with them and paying attention to patients as individuals. Demonstrations of kindness and respect meant that instead of clinicians being “brutish” with patients, perhaps “they should just talk—ask about their family” (M09, Hispanic, Spanish-speaking birthing parent). The participants wanted their healthcare team to ask them how their day was going and for patients’ mental health to be actively supported. Quality practice included patients being asked “if everything's okay” and “if your needs have been met” as part of making sure patients feel “secure, safe, and like they know everything and are on top of it” (M54, non-Hispanic Black birthing parent). Patient safety was also addressed in relation to healthcare team hygiene in the context of the COVID-19 pandemic and adherence to clinical care protocols (e.g., antibiotic administration after surgery).

Support for maternal coping was reported as critical to patient quality of life but typically absent from clinical practice. A non-Hispanic Black birthing parent described their chronic pain and escalation of emotional distress thus:

“I think really what my health care providers should really ask their patients, really, really, is what is bothering you the most? Which issue is bothering you the most? Is it the migraines? Is it the back pain? What is bothering you the most? When it's bothering you, how do you cope with it? How are you dealing with it? How are you dealing with it mentally? ‘Cause a lot of people don't ask—a lot of physicians don't ask their patients, ‘You're in this type of pain on an everyday basis. How are you dealing with this mentally? How is it affecting you mentally?’ You know, because if you can figure out how it's affecting them mentally, then you can figure out how to treat them because you've got people that are in pain 24/7 and mentally they're depressed and they're not even coming out the house. The only time they're coming out the house is to see their physician, and then they tell their physician, ‘I'm in pain and this is what's going on,’ and then the physician changes the medicine or ups the dosage. But they never asked, ‘Mentally how are you doing? How are you coping with this? How are you dealing with this? Are you still going out? Are you still socializing or are you in the house all day? Are you underneath the covers?’… I'm telling you. If you look at a symptom and you work with trying to cure one of those symptoms, you have to figure out what the problem is. Trust me. It's gonna pop up. So pay attention….” (M31).

Recommended patient-reported experience metrics were all about patients feeling heard, comfortable, and safe—meaning that they are taken care of as a whole person and that they are not confused or worried about their care. The participants wanted to be “aware of all the [health care service] options … to make sure that we feel empowered to make decisions” (M30, non-Hispanic white birthing parent). Indicators of quality care also included the extent to which patients were meeting their goals, feeling happy with their care, and whether anything could be done better. A non-Hispanic Black birthing parent suggested asking whether people's needs have been met: “Making sure the patient feel secure, safe, and like they know everything and are on top of it” (M54). In addition, postpartum visit attendance was offered as a patient healthcare service utilization metric to indicate the quality of their previous perinatal care.

### Timing—ongoing opportunities for patients to provide feedback

Participants described quality care as a system in which healthcare team members “check-in” with patients as part of an ongoing, direct dialog. At the same time, the participants noted that asking someone about their care directly will “not guaranteed to get an honest response” (M55, non-Hispanic Native American birthing parent). Birthing parents wanted opportunities to provide feedback on perinatal care experiences triggered soon after encounters and for these responses to be shared with someone who was not a part of their healthcare team. Opportunities for patient feedback and healthcare responses “along the way” were important for multiple reasons. A participant recommended that the quality of perinatal care be assessed after every healthcare encounter “because it could be different from visit to visit” (M52, non-Hispanic multirace birthing parent). Furthermore, knowing issues “when it's fresh and when it's happening” and dealing with it in real time was preferred instead of writing a review or complaint later “cause sometimes things can fester and then the report can turn into something much bigger because their emotions have now sat on it and it's made them feel worse or less” (M22, non-Hispanic Native American birthing parent). Timing consideration for meaningful measurement became a complex factor because of patient fatigue. The participants described their fatigue as being substantial in the postnatal unit and following hospital discharge, limiting patient ability to offer feedback on those healthcare services.

### Process—establishing a robust, universally accessible, and safe feedback system

The patients desired a “serious platform” with accessible methods for all birthing parents to be able to convey nuanced accounts of their perinatal care. The participants described current healthcare evaluation surveys as helpful for efficient administration, standardized patient responses, and comparisons. However, an Asian birthing parent reported limited engagement with surveys and expressed doubts about their utility because they were overly clinical:

“I mean, these patient satisfactory surveys that you get, no one fills them out. I think the questions are worded generally, they're worded so that it's almost like a black and white clinic kind of answer. And in most cases, it's really not a black and white kind of answer, and it really involves more digging, which you're not going to get through an electronic survey. I understand the need to do that because you need numbers, and you need to be able to standardize your answers, and it's an easy way to collect information. I think it's going to require more than that. I think what you're doing, for example, having these in-depth interviews and questions, almost like you're asking the same question ten times but in ten different ways. But you're really getting to the meat of things that way. So, I think that is one way to really assess if someone is getting the care that they needed, and if they didn't then you would find out why.” (M27).

Similarly, a Hispanic Spanish-speaking birthing parent said it takes “more than a survey … to truly realize what the patient feels” (M39). Structuring multimethod (e.g., surveys or interviews) feedback opportunities for people to opt in as they like was described by another Hispanic Spanish-speaking birthing as promoting patient autonomy:

“Personally, with me [measuring the quality of perinatal care] is what you're already doing now [through the research interview]. They arrive and they introduce themselves, they tell you their names, and their job, and what they do. They ask you how you're doing. And they say, ‘I'm here for this reason and this and this.’ For me it's what they explain, and they don't just come to say, ‘I'll give you this.’ And you don't know what it is. No. They go through the process and explain for you to understand. And if you don't want to do it, they say, ‘Okay. You don't have to take it if you don't want to. You don't have to listen to this if you don't want to.’” (M29).

In addition to having comprehensible information about the potential to volunteer feedback, the participants wanted to know whether their perspectives were welcomed as part of healthcare system strengthening. A non-Hispanic Native American birthing parent said that there should be an opportunity to establish a supportive “circle of safety” for patients through “… dialogue with patients about letting them know that they can be honest and open … what you feel and what's happening or who you're encountering or what you're encountering” (M22). The need for clear communication around patient feedback was important to reduce patient worry about potential negative clinician response: “You never know how someone can respond to you” (M22, non-Hispanic Native American birthing parent). There was a specific concern about possible “retaliation” (M12, non-Hispanic Black birthing parent). As part of building trusting relationships, the participants desired institutional responses to patient feedback.

## Discussion

In this qualitative study with diverse birthing parents in the Southeastern United States, we asked participants about how we, collectively, could know whether families were receiving quality perinatal healthcare. Their responses offer insights into what, when, and how we might appropriately measure healthcare service delivery and impact. The participants desired a robust and protected clinical feedback cycle, with multimethod opportunities for sharing healthcare practices and experiences throughout their perinatal journeys—not only retrospective survey assessments. They also sought institutional responses to feedback, as part of healthcare system accountability for the provision of ethical and effective perinatal services. Instead of a one-off, one-way measurement, the patients recommended an integrated cycle of communication for achieving quality perinatal care. The results suggest that public posting of healthcare evaluation, such as the one with Hospital Consumer Assessment of Healthcare Providers and Systems survey results in the United States, may be insufficient for promoting individual participation in clinical feedback opportunities. Instead, we might consider proactively communicating the ways in which patient input is heard and addressed by healthcare systems, for shared understanding that patient feedback is safe and worthwhile.

Participant responses in this study focused on aspects of perinatal care and experiences they desired. This positive framing is notable. The findings suggest that birthing parents conceptualize measurement of quality care focused on their feelings of being taken care of, not only on the absence of harm. Mistreatment of birthing people through perinatal care is unacceptably prevalent in the United States (13, 14) and beyond (15, 16). In addition to eliminating a “power-over” mentality in perinatal care and its manifestations (17), our findings underscore the importance of healthcare team–patient connection and collaboration. Previous research on welcoming, supportive perinatal care lists multilevel aspects of services that patients reported as helpful and impactful (18–20). The previous findings and our results identified the components of quality healthcare team member conduct that included an upbeat attitude, referring to patients by their names, and other feasible yet currently variable components of care. Maternity care, particularly postpartum services, is an opportunity to not only accommodate patient safety but also uplift birthing parents and families (7).

The study results are limited in their transferability to other settings because of the single-site design of the study. Other medical centers and obstetric facilities in the United States and beyond could partner with funded patient advisory councils and healthcare team members with protected time to consider the metrics, timing, and process recommendations offered through this research. Our results suggest that patient perceive quality care as multifaceted, which is probably more than the sum of its parts. A range of healthcare practices and patient experiences mattered to participants. At the same time, asking about measurement is not the same as comprehensively defining the components of quality care, which is an important area for future work. Perinatal care is a sacred life event and quality measurement therefore must extend beyond assessing user satisfaction and tracking health outcomes. National initiatives such as the Birthing Friendly Hospital designation could involve partnership with diverse patients, including those from various geographic locations, those with different health conditions, varied insurance coverage, different ages, those speaking different languages, and those with perinatal health outcomes, to develop tools and processes for widespread implementation and sustainment.

Approaches to measurement are critical, as feedback offers insight into the strengths of, and areas of opportunity for, quality care. Careful attention is necessary because the participants expressed worry not only about antagonizing clinicians, but also about the fear of retaliation. Patient worry about how information might be used against them and self-protective behavior in perinatal care are consistent with findings in previous research (19, 21). A continuous review of feedback utilization disaggregated by ethnicity, race, and other factors and subsequent follow-through to improve access is important. The people who are most marginalized may particularly not have the time, energy, or space to participate in offering feedback. In addition to health outcomes, emotional safety—before, during, and after healthcare encounters—is a critical component of patient safety (22).

Alignment and integration of patient-focused measurement is highly promising for improving the quality of perinatal care. At the same time, caution should be exercised to recognize scores as indicators of the ways people are being treated as part of a cycle to respectful care (23). Ratings on any quality measure, no matter how meaningful, are not sufficient when considered as a checklist. Healthcare systems are comprised of people, and all stakeholders should have opportunities to offer their insights into practice strengths and opportunities for improvement. Alongside patient accounts, future work could investigate the ways that the perspectives of frontline perinatal healthcare team members are leveraged for taking meaningful measures and initiatives. Creese et al. (24) identified organizational “deafness, disconnect between managers and frontline staff, and denial of the narratives and issues raised” as barriers to integrating “employee voice” in hospital administration. These researchers identified similar barriers to measuring quality care as our findings, including the current one-directional nature of communication and subsequent disengagement with the feedback system. For patients to be well, and because of the inherent value of all individuals and of meaningful work, healthcare team members need access to conditions to also enable them to thrive.

## Conclusion

In this study, it was found that birthing parents expressed desire for their perinatal healthcare experiences to be understood. They outlined meaningful metrics, timing, and process considerations to establishing a “circle of safety.” Strengthening opportunities for feedback and ensuring accountability are ways to demonstrate respect, promote patient autonomy, and build trusting relationships through quality care. Creating and communicating assurances of confidentiality, given that patients are aware that they may see the same clinicians in the future, is an important and sobering component of improving healthcare services.

## Data Availability

The raw data supporting the conclusions of this article will be made available by the author without undue reservation.
